# Efficacy and Safety of 1% Atropine on Retardation of Moderate Myopia Progression in Chinese School Children

**DOI:** 10.7150/ijms.39365

**Published:** 2020-01-01

**Authors:** Qin Zhu, Yang Tang, Liyun Guo, Sean Tighe, Yuan Zhou, Xiaofan Zhang, Jieying Zhang, Yingting Zhu, Min Hu

**Affiliations:** 1 Department of Ophthalmology, The Second People's Hospital of Yunnan Province (Fourth Affiliated Hospital of Kunming Medical University); Yunnan Eye Institute; Key Laboratory of Yunnan Province for the Prevention and Treatment of ophthalmology (2017DG008); Provincial Innovation Team for Cataract and Ocular Fundus Disease (2017HC010); Expert Workstation of Yao Ke (2017IC064), Kunming 650021, China; 2 Tissue Tech, Inc., Ocular Surface Center, and Ocular Surface Research & Education Foundation, Miami, FL, 33126 USA

**Keywords:** atropine, myopia, therapeutic effect, Chinese children

## Abstract

**Background:** To evaluate the long-term efficacy and safety of topical 1% atropine for retarding moderate myopia.

**Methods:** A randomized, controlled study evaluating atropine and placebo in 660 Chinese children. Patients received drops q1month for 24 months, then q2month for 12 months, followed by no drops for 12 months. Spherical equivalent, axial length, intraocular pressure and atropine-related side effects were examined at 6, 12, 24, 36 and 48 months for all children.

**Results:** Spherical equivalent, myopic progression, axial length augmentation, and progression rate were significantly reduced in the atropine group than those in the placebo group (all P<0.05), indicating that 1% atropine effectively retarded myopia. Moreover, myopic rebound and adverse effects of 1% atropine were eliminated by gradual withdrawal and elimination of 1% atropine. Furthermore, pupil size, near visual acuity, and amplitude of accommodation returned to pretreatment levels after withdrawal of atropine.

**Conclusion:** Topical 1% atropine periodically and alternatively in phase I with gradual reduction in phase II and final withdrawal in phase III may effectively improve atropine efficacy, retard moderate myopia, reduce atropine side effects, minimize myopic rebound, and increase compliance of children simultaneously.

## Introduction

Myopia is a major public health concern that has become increasingly common 1. The overall prevalence of myopia has increased from 79.5% to 87.7%, with a significant increase of moderate myopia (38.8% to 45.7%), severe myopia (7.9% to 16.6%), and terminal myopia (0.08% to 0.92%) 2. In China, the prevalence of myopia is 27% in primary school students and as high as 81% in high-school students 3, 4. Unfortunately, severe myopia has become one of the main causes of untreatable vision loss throughout the world, often due to its irreversible complications, such as retinal detachment, retinal break, macular degeneration, choroidal neovascularization and glaucoma 5-8. The risk of these complications increases with the severity of myopia and the early onset of myopia in childhood is associated with the severity of myopia in the adult life 9-12.

The most common form of myopia worldwide is secondary to elongation of the axial length of the eye, termed axial myopia. Axial lengthening begins in childhood and progresses remarkably during the adolescent growth period. In this period, the risk of myopia-related complications increases proportionately with axial length. Therefore, the most effective strategy to reduce myopia-related complications is to delay myopia progression during childhood 13, 14.

At present, options for retarding myopia progression include progressive addition of executive bifocal spectacle lenses 15-17, peripheral defocusing lenses 18, contact lenses 19, overnight orthokeratology 20-22, multifocal soft contact lenses 23, outdoor activities 24, and pharmacological agents 25. Unfortunately, long-term outcomes using bifocals, progressive addition lenses, and contact lenses have been unsatisfactory in myopia control (reviewed in 18). Alternatively, pharmacological intervention using atropine has gained recent interest based on results from the Atropine for the treatment of childhood myopia (ATOM) study. 26-31 This double-blinded study showed topical atropine was well tolerated and effective in slowing the progression of low and moderate myopia and ocular axial elongation in Asian children. Despite these satisfactory results, a proper treatment regimen for effective treatment of myopia by atropine has not been established. In addition, there is concern of possible long-term side effects of 1% atropine eye drops, including phototoxic effects on the retina and lens 32, near vision blurring, photophobia, allergic reaction, and myopic rebound after the treatment is discontinued 33.

Previously we evaluated if lowering the concentration of atropine from 1% to 0.01% would result in safer outcomes. 34 Unfortunately, the lower concentration was not effective in preventing myopia progression. Therefore, the present study was designed to evaluate a modified dosing regimen of 1% atropine in decreasing side effects and retarding myopia progression. Our results suggest that this issue can be satisfactorily resolved by using 1% atropine periodically and alternatively in phase I, with gradual reduction in phase II and elimination in phase III. Such a treatment may not only significantly and effectively slowdown the progression of myopia, but also profoundly reduce the side effects of atropine and minimize myopic rebound.

## Materials and Methods

### Design

The design was an effectiveness study, prospective and clinic-based. This study followed the tenets of the Helsinki Declaration on ethical principles for medical research involving human subjects and was approved by the Committee for Protection of Human Subjects (CPHS) of Yunnan Eye Research Institute Review Board, the Second People's Hospital of Yunnan Province, China. Children were recruited in the hospital from December 2014 to December 2018. The informed written consent was obtained from all subjects prior to participation in the study after the nature of the study and the possible outcomes were disclosed.

Assignments to the control (placebo) and the experimental (1% atropine) groups were allocated with concealment according to a computer-generated randomization list after eligibility criteria were verified. The children had, therefore, an equal probability of assignment to either the experimental group or the control group. As a result, 330 children from the experimental group and 330 children from the control group were enrolled in this investigation. A study number was issued to each child. The plan was adhered strictly to the experimental design specified below in the entire trial.

### Criteria

In the meta-analysis, the subjects met the following criteria: (1) Initial myopic spherical equivalent ranged of -2.0D to -8.00D, and astigmatism ≤ 1.0D. (2) Recruited children age was between 6 to 12 years, willing to participate in the entire 4-year trial. (3) SE progression rate was ≥1D/Year in the last year. (4) Binocular function and stereopsis were normal. (5) Intraocular pressure was normal (IOP<21 mmHg). (6) Ability against cycloplegia and mydriasis was a must.

The exclusion criteria were as follows: (1) Children with ocular diseases, such as amblyopia, strabismus, congenital cataract, glaucoma, corneal scar, optic neuropathy, traumatic ocular injury, uveitis, or ocular tumor were excluded. (2) Children with history of any ocular surgeries were excluded. (3) Any systemic diseases or conditions that could affect visual function and development, including diabetes mellitus and/or chromosome anomaly, were excluded. (4) Previous or current use of contact lenses, bifocals, progressive addition lenses, or other forms of treatment, including atropine, for the control of myopia were excluded.

Only those patients whose families formally confirmed 100% compliance to the treatment and who had more than 1 year follow up (during this year's follow-up, myopia progression >1D/year) were included in the study. Both parents and children are required to fill in questionnaire surveys, including the time and the quantity of eye drops received, the adverse events and the duration.

### Subjects and Experiments

Six hundred and sixty myopic school children met the above criteria were included in the study. The experimental group, including 330 myopic children in phase I, according to their chart records, received topical 1% atropine eye drops (Xingqi Pharmaceutical Co., Ltd. China) at bedtime once a month (one eye received treatment at day 1, the other eye received treatment at day 16) for 24 months. Spherical equivalent (SE) and axial length (AL) were determined for the children at 6-month interval. Intraocular pressure (IOP) and potential atropine-related side effects were evaluated at 6-month interval. In phase II, the frequency of the medication was reduced to once two-months, for 12 months. The examinations specified above were also at 6-month interval. In phase III, no atropine was applied to the children for 12 months (withdrawal of atropine). During the three stages, 330 children received the corresponding treatment of saline (placebo) and frame glasses as the controls. All children in the control group were examined similarly as those in the experimental group.

During this study, photo chromatic progressive addition lenses with ultraviolet protection were used to minimize the photophobia, glare, and potential toxicity to the retina and lens due to long-term dilation and exposure to ultraviolet light.

Cycloplegic autorefraction was obtained 45 minutes after administration of 1% cyclopentolate eye drops, performed by investigators who were trained in the study protocols by Topcon auto refractor KR8900 (Topcon, Tokyo, Japan). Uncooperative children refractive error was determined by the experienced orthoptist (JRP) performing retinoscopy with a Heine beta 200 retinoscope (Heine Optotechnik, Herrsching, Germany) and the lenses according to the standard protocols. Spherical equivalent was calculated using the standard formula: (SE=sphere+1/2 cylinder). After cycloplegic refraction, axial length was measured by IOL Master 500 (Carl Zeiss MEDITEC IOL-master, Jena, Germany). IOPs were obtained via noncontact tonometry (Nidek Co. Ltd, Tokyo, Japan). Initial ocular investigations at our hospital included slit lamp biomicroscopic examination for anterior segment, direct or indirect ophthalmoscopic examination for vitreous, retina and optic disc evaluation. All control cases were matched with the experimental cases in terms of age, sex, and initial SER (±0.50 D), to avoid potential bias. The control cases received the same ophthalmic examinations as the experimental subjects. At each follow-up visit, all children were required to change their glasses if the SE progression rate ≥0.5D.

During each visit, children and parents were given an open-ended opportunity to report any medical illness or side effects, including any symptoms related to allergy, blurred near vision, glare, or visual loss. The details of such reports were documented.

### Statistical Analysis

All statistical analyses were based on the intention-to-treatment principle and performed using SAS software version 9.13 (Cary, NC). For continuous variables such as age, the duration of follow-up, and spherical equivalent refraction, analysis of variance and the Student's t-test was used to determine the statistical significance between the groups. For continuous variables, such as unaided visual acuity, spherical equivalent refraction and axial length, analysis of variance of repeated measure data was used to determine the statistical significance between the groups. The χ^2^ test was used to compare the categorical variables. Throughout the study, P<0.05 was considered as statistically significant.

## Results

Between December 2014 and December 2018, 660 children were randomly enrolled in the study, of which 330 from the experimental group and 330 from the control group. At the initial pretreatment visit, there was no significant statistical difference between the two groups in mean sex, age, spherical equivalent, axial length, biometric characteristics and parental myopia (all P>0.05, Table 1-3).

A total of 570 (86.4%) subjects completed the 4-year study. Of 90 cases who did not, 22 were from the control group and 68 were from the experimental group. The most frequent reasons for discontinuation in the experimental group were photophobia and reading problems within the first month. As the children continued to take the medication of 1% atropine for more than three months, the adverse reactions became very mild, according to the questionnaire. In addition, the reading difficulties of most children were significantly reduced after three months of medication and the rate of interruption greatly decreased. Only those patients who did not miss the medication during the trial period were included in the analysis.

At the end of phase I (i.e. 24 months), the mean progression of myopia in 1% atropine group was significantly reduced when compare to that in the control group [(-0.27±0.81D)/Year vs (-1.29±0.13 D)/Year, P<0.05, Table 3]. In addition, axial length increase in the experimental group was also significantly decreased when compared to that in the control group [(0.11±0.13 mm)/Year vs (0.41±0.19 mm)/Year, P<0.05, Table 3]. At the end of the second phase (25-36 months), the mean progression of myopia in the 1% atropine group was significantly diminished when compared to that in the control group [(-0.31±0.29 D)/Year, vs (-0.80±0.66 D) /Year P<0.01, Table 3]. The mean increase in axial length in the atropine group was profoundly reduced when compared to that in the control group [(0.14±0.09 mm)/Year vs (0.39±0.14 mm)/Year, P<0.001, Table 3]. Furthermore, at the end of phase III (36-48 months), myopia progression in 1% atropine group was significantly decreased when compared to that in the control group [(-0.41±0.23 D)/Year vs (-0.75±0.64 D)/Year, P<0.05, Table 3]. The mean increase in axial length in the experimental group was also significantly diminished when compared to that in the control group [(0.19±0.13 mm)/Year vs (0.40±0.16 mm)/Year, P<0.001, Table 3].

Interestingly, at the end of the 4-year study, the final SER in 1% atropine group was significantly reduced when compared to that in the control group [(-4.96±1.22 D) vs (-7.28±1.26 D), P<0.001, Table 3]. In addition, the final AL in the experimental group was significantly reduced when compared to that in the control group [(25.48±0.29 mm) vs (26.59±0.20 mm), P<0.01, Table 3]. Furthermore, the final mean myopia progression per year (mean myopia progression rate) in the experimental group was significantly decreased when compared to that in the control group [(-0.29±0.17 D) vs (-0.89±0.44 D), P<0.05, Table 3]. Finally, all 288 (100%) children in the control group had faster myopia progression, defined as myopia progression greater than -0.5 D per year, in contrast to 46 (16.3%, out of 282) children in the experimental group.

Meanwhile, at the end of follow-up, pupil size and near visual acuity returned to pre-atropine levels in both groups. In addition, the amplitude of accommodation and near visual acuity also returned to the pretreatment levels. Furthermore, throughout the follow-up period, no serious adverse events related to atropine were noted. Adverse events in children who maintained and ceased therapy in treatment group were: photophobia 205/330 (62.12%), blurred near vision 65/330 (19.70%), allergic reaction 3/330 (0.9%) eye irritation 61/330 (18.5%), and infections (conjunctivitis, blepharitis) 18/330 (5.451%) (Table 4).

The reasons for the withdrawal from the trial were: forget (12.0%), photophobia (10.0%), eye irritation (8.2%). No patients complained distention of eyes, ocular redness, or foreign body sensation, and so forth. No changes were observed in IOPs, crystalline lenses, optics disk, or macula following atropine administration.

According to the records from the parents, the blurred near vision disappeared 4.6 days in average after atropine applications. Because atropine was used in one eye every month instead of both eyes, mild photophobia and blurred near vision did not affect children's daily study and life. Atropine subjects had no-significantly myopic rebound during recovery phase III.

## Discussion

Atropine, an alkaloid derived from Atropa belladonna, acts as an antagonist against the nonselective competitive muscarinic acetylcholine receptors. Topical atropine has previously been shown to delay myopic progression and axial elongation 26 in a dose-dependent manner 28, 34. The 1% atropine group has been demonstrated to be the most effective (78%) followed by 0.5% group (75%), 0.1% group (70%), and 0.01% group (50%) 28, 34. Unfortunately, long-term side effects have been attributed to 1% atropine eye drops 33. Therefore, we carefully designed this clinical study to evaluate 1% atropine drops only once a month, eye to eye alternatively in the first two years as a treatment period, every 2 months, eye to eye alternatively in the third year as a transition period, and withdrawal of atropine eyedrops in the fourth year in Chinese children to determine if this decreased side effects but maintained effectiveness in retarding myopia progression.

At 2 years, our results showed the mean myopia progression in the atropine group was significantly reduced, when compared with that in the control group [-0.45 D (-0.225D/Year) vs -1.94 D (-0.97D/Year)]. Axial length increase in the experimental group was also significantly decreased, when compared to that from the control group [(0.25±0.31 mm) vs (0.81±0.39 mm)]. These results indicate that myopia progression and axial length increase were reduced by 76.8% and 69.1% respectively, similar to the reports that daily use of 1% atropine slowed down myopia progression and axial elongation by approximately 78% and 70% in previous trials 28, 29, 31, 34, 35. Thus, we established that 1% atropine was equally effective in reducing myopia progression when used daily or monthly.

Although 1% atropine was effective in slowing myopic progression as previously shown in other studies as well (Tran HDM et al, 2018), a significant rebound phenomenon of myopia has been noted.^36^ To minimize such a rebound, we reduced the frequency of 1% atropine from once a month to once every two months from years 2 to 3, aiming to prevent myopia rebound caused by the withdrawal of atropine abruptly. Interestingly, our results demonstrated that the mean progression of myopia in the atropine group was significantly reduced than that in the control group [(-0.31±0.29 D)/Year vs (-0.80±0.66D) /Year)], suggesting that reduced frequency of 1% atropine use is indeed effective to significantly decrease myopia rebound.

Because sudden cessation of atropine drops and high dose (for example, 1%) has been associated with the rebound in myopia progression 37, 38, we chose to add one-year withdrawal period (phase III), aiming at reducing such a rebound. As expected, our results showed that after withdrawal of 1% atropine for 1 year, the mean progression of myopia in our 1% atropine group was significantly decreased when compared to those from 1%, 0.5%, 0.1% atropine groups in which atropine eyedrops were used daily and withdrew abruptly [(-0.41±0.23 D)/Year vs those atropine used daily and withdrew abruptly 1% group (-1.14±0.80 D), 0.5% group (-0.87±0.52 D), 0.1% group (-0.68±0.45 D) 2. In addition, myopic rebound in our observation was reduced by two third, when compared to that reported previously 28, 34, 37, 38, suggesting that our method achieved a significant greater reduction in both progression of childhood moderate myopia and myopic rebound by gradually withdrawing the frequency of atropine eye drops in phase III. Our results showed that over the 4-year period, ~68% reduction in mean progression of myopia and 65% decrease of axial growth in the experimental eyes were achieved, when compared to those in the control group. Our study also demonstrated that 1% atropine treatment was well tolerated generally, with no serious adverse effects.

Based on our results, we recommend the following guidelines in controlling myopia: First, identify and discuss the risk factors with the patients and provide lifestyle advice, such as increase of the outdoor time. Second, start intervention with atropine 1% and photo chromatic glasses in high risky patients. Third, perform regular follow-up examinations including visual acuity, reading acuity, cycloplegic refraction, and axial length. Finally, adjust clinical treatment regimen as we have recommended. When SE and axial length remain stable for a period of 12 months, gradually taper the atropine concentration to naught.

## Conclusion

In conclusion, our study shows for the first time that our established procedure, treatment of one eye at one time point and the other eye at the other time point with 1% atropine to achieve frequency of once a month in the first two years and gradually withdrawal of atropine to once two months in the third year with a recovery fourth year can effectively retard the progression of moderate myopia with a significant reduction in myopic rebound in school children. Our method provides a remarkable choice for effectively treatment of myopia in school children, in our daily clinical practice anywhere in the world, thus, preventing mind and moderate myopic children from becoming severe myopia patients later in life.

## Acknowledgement s

This work is supported by the National Natural Science Foundation of China, Grant No. 81560168.

### Author Contributions

Q Zhu, Y Tang and LY Guo have contributed to experimental design, performance of experiments and manuscript writing. S Tighe has contributed to manuscript organizing, writing and English editing. Y Zhou, XF Zhang and JY Zhang have contributed to performance of experiments. M Hu and YT Zhu have overseen the experimental design, performance and finalized the manuscripts.

## Figures and Tables

**Table 1:** Baseline Demographic Characteristics

Characteristics	Experimental Group (n =262)	Control Group (n =308)	P Value
Male/Female	130/132	156/152	0.22
Age	9.11±0.09	9.19±0.14	0.10
Initial SER, D	-3.82±0.44	-3.74±0.51	0.29
Axial Length	24.93±0.21	24.91±0.18	0.28

**Table 2:** Parental Myopia and Age

	Experimental Group	Control Group	P Value
SE of Mother	3.26±3.17	3.29±3.23	0.16
SE of Father	4.01±3.83	4.12±3.91	0.28
Age of Mother	27.91±5.68	28.12±5.09	0.10
Age of Father	28.71±6.87	28.81±6.47	0.43

**Table 3:** Spherical Equivalent and Axial Length over Time in Treatment Group and Control Group

	Experimental Group (n =262)	Control Group (n =308)	P Value
**Spherical Equivalent Refraction (SER)**			
Before Treatment (D)	-3.82±0.44	-3.74±0.51	0.30
6 Months after Treatment (D)	-3.91±0.35	-4.15±0.89	0.03
12 Months after Treatment (D)	-4.05±0.97	-4.79±0.82	0.020
18 Months after Treatment (D)	-4.11±0.80	-5.21±0.88	0.016
24 Months after Treatment (D)	-4.27±0.21	-5.68±1.03	0.011
30 Months after Treatment (D)	-4.41±0.93	-6.12±0.73	0.009
36 Months after Treatment (D)	-4.58±1.32	-6.59±1.10	0.006
48 Months after Treatment (D)	-4.96±1.22	-7.28±1.26	<0.001
**Myopic Progression Rate (PR)**			
Before Treatment	-1.28±0.81	-1.29±0.13	0.62
6 Months after Treatment (D/year)	-0.27±0.16	-1.01±0.49	<0.001
12 Months after Treatment (D/year)	-0.24±0.22	-0.98±0.90	<0.001
18 Months after Treatment (D/year)	-0.22±0.14	-0.91±0.61	<0.001
24 Months after Treatment (D/year)	-0.21±0.22	-0.89±0.23	<0.001
30 Months after Treatment (D/year)	-0.29±0.19	-0.82±0.14	<0.001
36 Months after Treatment (D/year)	-0.31±0.29	-0.80±0.66	0.008
48 Months after Treatment (D/year)	-0.41±0.23	-0.75±0.64	0.012
**Axial Length (AL)**			
Before Treatment	24.93±0.21	24.91±0.18	0.30
6 Months after Treatment (mm)	25.00±0.18	25.13±0.12	0.16
12 Months after Treatment (mm)	25.03±0.11	25.34±0.08	0.126
18 Months after Treatment (mm)	25.10±0.15	25.57±0.14	0.018
24 Months after Treatment (mm)	25.18±0.21	25.72±0.17	0.009
30 Months after Treatment (mm)	25.26±0.18	25.98±0.13	0.002
36 Months after Treatment (mm)	25.31±0.14	26.18±0.14	0.001
48 Months after Treatment (mm)	25.48±0.29	26.59±0.20	<0.001
**AL Progression Rate (PR)**			
Before Treatment	0.41±0.27	0.42±0.26	0.9
6 Months after Treatment (mm/year)	0.11±0.13	0.41±0.19	<0.001
12 Months after Treatment (mm/year)	0.12±0.10	0.40±0.06	<0.001
18 Months after Treatment (mm/year)	0.12±0.16	0.40±0.11	<0.001
24 Months after Treatment (mm/year)	0.12±0.10	0.39±0.19	<0.001
30 Months after Treatment (mm/year)	0.13±0.06	0.39±0.04	<0.001
36 Months after Treatment (mm/year)	0.14±0.09	0.39±0.14	<0.001
48 Months after Treatment (mm/year)	0.19±0.13	0.40±0.16	<0.001

**Table 4:** Adverse Events in Children Who Maintained and Ceased Therapy in the Experimental Group

	Maintained Therapy (n =262)	Ceased Therapy (n =68)	Total (n=330)
Photophobia	154/262 (58.78%)	51/68 (75.0%)	205 (62.12%)
Blurred Near Vision	56/262 (21.37%)	9/68 (13.24%)	65 (19.70%)
Headache	36/262 (12.8%)	3/68 (4.41%)	39 (11.82%)
Allergic Reaction	3/262 (1.1%)	0/68 (0%)	3 (0.9%)
Eye Irritation	59/262 (22.52%)	2/68 (2.94%)	61 (18.5%)
Infections (Conjunctivitis, Blepharitis)	18/262 (6.87%)	0/68 (0%)	18 (5.45%)
